# Tracking Subtle Stereotypes of Children with Trisomy 21: From Facial-Feature-Based to Implicit Stereotyping

**DOI:** 10.1371/journal.pone.0034369

**Published:** 2012-04-04

**Authors:** Claire Enea-Drapeau, Michèle Carlier, Pascal Huguet

**Affiliations:** Laboratoire de Psychologie Cognitive, Aix-Marseille University and Centre National de la Recherche Scientifique, Marseille, France; French National Centre for Scientific Research, France

## Abstract

**Background:**

Stigmatization is one of the greatest obstacles to the successful integration of people with Trisomy 21 (T21 or Down syndrome), the most frequent genetic disorder associated with intellectual disability. Research on attitudes and stereotypes toward these people still focuses on explicit measures subjected to social-desirability biases, and neglects how variability in facial stigmata influences attitudes and stereotyping.

**Methodology/Principal Findings:**

The participants were 165 adults including 55 young adult students, 55 non-student adults, and 55 professional caregivers working with intellectually disabled persons. They were faced with implicit association tests (IAT), a well-known technique whereby response latency is used to capture the relative strength with which some groups of people—here photographed faces of typically developing children and children with T21—are automatically (without conscious awareness) associated with positive versus negative attributes in memory. Each participant also rated the same photographed faces (consciously accessible evaluations). We provide the first evidence that the positive bias typically found in explicit judgments of children with T21 is smaller for those whose facial features are highly characteristic of this disorder, compared to their counterparts with less distinctive features and to typically developing children. We also show that this bias can coexist with negative evaluations at the implicit level (with large effect sizes), even among professional caregivers.

**Conclusion:**

These findings support recent models of feature-based stereotyping, and more importantly show how crucial it is to go beyond explicit evaluations to estimate the true extent of stigmatization of intellectually disabled people.

## Introduction

Trisomy 21 (T21) or Down syndrome is the most frequent genetic disorder associated with intellectual disability, affecting between 1.0 and 2.2 of every 1000 live births according to statistics on prenatal testing and selective abortion [1]–[4]. Because this chromosomal disorder is also associated with various health problems (e.g., hypotonia, congenital heart defects, gastrointestinal diseases) and distinctive physical stigmata (e.g., round face, epicanthal fold, oblique lid axis, flat nasal bridge), persons with T21 are at high risk of being rejected socially. Not only are the common societal reactions unfavorable in terms of rates of fetal termination and abandonment [2], [5], but those who live with T21 are likely to be stigmatized by other people [6]. This is a critical issue, because stigmatization is one of the greatest obstacles to the successful integration and development of people with intellectual disabilities.

Although research in this area is very limited, it seems that persons with T21, especially children, are typically viewed as “friendly”, “affectionate”, and “happy” (e.g., [7]–[9]); with positive personality traits prevailing over the negative ones (e.g., “mentally retarded”). This positive stereotype, however, coexists with ambivalent attitudes about the integration of these children into regular schools (e.g., [8], [10], [11]). Based on a recent survey, Pace et al showed that whereas 45% of adult respondents (*N* = 5399) from the general (U.S.) population agreed with inclusive education for students with T21, 25% disagreed (considering for example that such students are “distracting”) and 29% abstained [11]. Likewise, about one-third of adolescent respondents (*N* = 1704) reported they were not willing to work on a class project or spend time outside of school with a student with T21. Although these negative attitudes are not majority rule, and can even be negligible in people who have relationships with persons with T21 [11]–[13], they may very be the trees that hide the forest.

Of particular interest here, all published studies on the social perception of intellectually disabled people, including those with T21, have measured attitudes and stereotyping solely at the explicit level. This is an important limitation, because social-desirability attitudes may interfere with the measurement of people's responses at this level. We are not suggesting that responses at the explicit level are deliberate lies that people use to cover up their implicit attitudes and convictions, but that it may not be sufficient to focus on consciously-accessible evaluations because people may be unaware of, or unwilling to report, all of their thoughts and feelings. There is ample evidence that attitudes and stereotypes may be used automatically, and thus quickly and effortlessly, without conscious awareness, and yet may influence perception, judgments, or actions even against a person's intentions [14]–[16]. For example, people who display explicit overt beliefs in the equality of the races may implicitly associate positive attributes (e.g., pleasant words) with White more than with Black persons (or more negative attributes with Black than with White persons). These automatic biases can be found even when pictures of equally unfamiliar Black and White people are used as stimuli, and when differences in stimulus familiarity are statistically controlled (e.g., [17]). There is also evidence that implicit evaluations explain variance in behavior over and above that explained by explicit evaluations (e.g., [18], [19]). Thus, by focusing exclusively on explicit (controlled) evaluations, past research on the social perception of intellectually disabled individuals has most certainly fallen short of revealing the whole picture.

### The present study

The present study investigated subtle stereotyping of children with full T21 likely to arise at both levels, i.e., explicit and implicit. First, we looked into whether the explicit evaluations of children's personality can be modulated by the degree to which their facial features are perceived as distinctive of T21. Because facial features can be powerful cues to category membership (e.g., when facial features are associated to racial categories), category-based stereotyping may ensue, as also suggested by standard stereotyping models [20]–[23]. Feature-based stereotyping may actually operate both between and within categories (e.g., [24]–[26]), resulting in greater stereotyping of people with a larger number of distinctive features of the category in question. Blair et al found that people with a greater number of Afrocentric facial features were presumed more likely to have traits that are stereotypic of African Americans than people with a smaller number of Afrocentric features [24]. Likewise, Blair et al found that prisoners with a greater number of Afrocentric facial features received more severe sentences than people with a lesser number of Afrocentric features [25]. In subsequent research [26], participants seemed to be able to control some aspects of race-based stereotyping, but appeared unaware of and unable to control (within-race) stereotyping based on Afrocentric features.

Here we suggest that children with T21 may also be subject to stereotyping based on their facial features, resulting in less positive judgments for those with features highly characteristic of this chromosomal disorder, compared to both their counterparts with fewer distinctive features and to typically developing children.

Second, we explored stereotyping of children with T21 using implicit association tests (IAT), a well-known technique whereby response latency is used to capture the relative strength with which some groups of people are associated with positive versus negative attributes in memory [15], [27]. In the current study, participants classified two types of stimuli: children's faces, and positive or negative personality traits, using two designated keys. We predicted faster reaction times when photographed faces of typically developing children (hereafter referred to as TD pictures) and positive traits shared the same key while photographed faces of children with T21 (hereafter referred to as T21 pictures) and negative traits shared the other key. Put differently, we predicted slower reaction times for the opposite combinations of stimuli: TD pictures and negative traits (associated with the same key), and T21 pictures and positive traits (associated with the other key).

Finally, the present research also tested whether the expected effects (of explicit and implicit stereotyping) can be found in people who have relationships with children with T21. There is evidence that relationships with intellectually disabled persons promote positive attitudes toward them (e.g., [11], [28], [29]). Again, however, previous research has left open the question of whether these relationships have any effect beyond mere awareness and reflection. Implicit stereotypes may be so deeply embedded in our culture that they may be activated regardless of whether a person considers them to be valid or invalid, and they have indeed often been described as being difficult to change (e.g., [14], [30]). On this basis, we assumed that even people who have relationships with children with T21 (e.g., professional caregivers) may exhibit subtle forms of negative stereotyping about them.

Thus, here we investigate for the first time subtle stereotyping of children with T21 based on their facial features, using both explicit and implicit levels of investigation, in different social groups ranging from young adult students to professional caregivers working with intellectually disabled persons.

## Materials and Methods

### Participants

The participants were 165 adults including 55 students (undergraduates and graduates) not enrolled in a psychology course (*M_age_* = 20.6 years, range: 18–25; *M* = 12.9 years of schooling, range: 10–17), 55 persons from the general population (referred to as non-student adults, *M_age_* = 38.3 years, range: 18–64; *M* = 14.0 years of schooling, range: 11–17), and 55 professional caregivers working with intellectually disabled persons, mainly individuals with T21 (*M_age_* = 39.1 years, range: 23–62; *M* = 13.7 years of schooling, range: 11–16). Students were 18 years younger and reported on average less than one year of schooling compare to participants of the two other groups (that did not differ from each other, *p*s >.28). All gave their written informed consent to participate in the present research, presented as a study on the “face perception of people with trisomy 21”. The project obtained approval from the Ethics Committee of Aix-Marseille Univ. (Avis Carlier 18.11.09).

### Procedure

All participants started with two counterbalanced T21-IATs created for this study using photographed faces of TD children and children with full T21. All parents of children photographed for the present research gave their written informed consent. Whereas one IAT involved faces weakly typical of T21, the other involved faces strongly typical of T21 (as also determined by a pre-test with another subject sample as described in Text S1). The photographs used in the two IATs were standardized. They showed only a face with a neutral facial expression against a blue background. In each IAT, participants classified 12 pictures, 6 TD pictures (faces of 3 male and 3 female children) and 6 T21 pictures (faces of 3 male and 3 female children with T21), in one of two categories, “trisomy” versus “normal”. They also classified 12 traits, 6 positively valenced ones (e.g., affectionate) and 6 negatively valenced ones (e.g., stupid), in one of two trait categories, positive versus negative (see Text S2 for details). In the combined task blocks of each IAT, participants switched between classifying exemplars of one contrast (TD pictures vs. T21 pictures) and exemplars of the other contrast (positive vs. negative traits). In half of the combined task blocks, TD pictures and positive traits were mapped to one response (e.g., right key) and T21 pictures and negative traits were mapped to the other response (e.g., left key). The other half of the combined-task block reversed the response mappings (e.g., TD pictures+negative traits vs. T21 pictures+positive traits). The order of administering combined tasks was randomly counterbalanced across participants. Overall participants performed 312 trials (including practice trials) structured in 7 blocks. The design of the IATs is presented in Table S1. Participants were told that a word or a picture face will occur in the middle of the screen and they have to press a key as quickly as possible to put each of these words or pictures in one of two categories (‘Positive’ vs. ‘Negative’ and ‘Trisomy’ vs. ‘Normal’, for the words and pictures, respectively). They also learned that these categories would be displayed at the top of the screen along with the associated key. Finally, they were instructed that errors (pressing the wrong key) would be indicated by an ‘X’ at the center of the screen that would imply to correct their answer to continue. If T21 pictures are more strongly associated to a negative valence than are TD pictures at the implicit level, classification should be faster in the “TD pictures+positive traits vs. T21 pictures+negative traits” blocks than in the “TD pictures+negative traits vs. T21 pictures+positive traits” blocks.

At the end of the two IATs, participants made explicit evaluations of the same pictures (18 in all), via each of the 12 traits used previously, resulting in 216 ratings for each participant. Participants were instructed that pictures would be presented at the center of the computer screen along with a word (bottom of screen). They were asked to indicate spontaneously to what extent the word was appropriate to the picture, using Likert type scale (from 1: “strongly disagree” to 6: “strongly agree”). These evaluations were produced after the two IATs so as not to prime stereotype-related cognition during the implicit tests. Consistent with a myriad of studies on impression formation, most participants agreed willingly to attribute personality traits to unknown persons and none of them stopped the task [31].

## Results

### Explicit Stereotyping

A mixed ANOVA was conducted with trait valence (positive vs. negative) and target of evaluation (TD pictures, T21 pictures weakly typical of T21, T21 pictures strongly typical of T21) as repeated measures, and source of evaluation (students, non-student adults, and professional caregivers) as between-participant factor. Not surprisingly, this analysis yielded a huge effect of trait valence, *F*(1, 162) = 593.23, *p*<.001, *η^2^*_p_ = .79. Participants attributed more positive traits (*M* = 4.63, *SE* = .06) than negative traits (*M* = 2.17, *SE* = .05) to all faces (see Table 1).

**Table 1:** Agreement score to the descriptive traits as a function of source of evaluation and target of evaluation.

	Faces					
	Typically developing		Weakly typical of T21		Strongly typical of T21	
	Traits					
Source of evaluation	Positive	Negative	Positive	Negative	Positive	Negative
Students	4.87 (.58)	1.80 (.56)	4.48 (.76)	2.44 (.81)	4.33 (.88)	2.66 (.99)
Non student adults	4.75 (.70)	1.90 (.65)	4.17 (.87)	2.59 (.90)	4.09 (.91)	2.82 (.94)
Professional caregivers	5.10 (.66)	1.50 (.44)	4.91 (.74)	1.86 (.68)	4.98 (.69)	1.93 (.70)

*Note*. Standard deviations are shown in parentheses.

**Figure 1:**
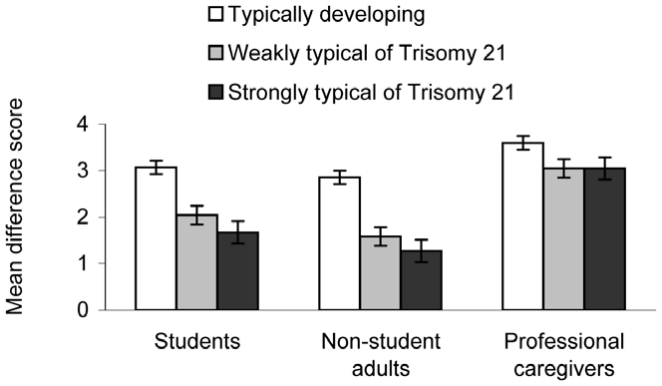
Explicit judgments. Mean difference score (positive traits minus negative traits) on explicit judgments as a function of target of evaluation and source of evaluation. Error bars represent ±1 SEM.

This difference favoring positive traits at the explicit level was modulated by both the target and the source of evaluation, *F*(4, 324) = 8.49, *p*<.001, *η^2^*_p_ = .09 (See Figure 1). This interaction was examined via two Helmert contrasts for each source of evaluation, with the difference in the trait valence score as the dependent variable. The first contrast compared the TD pictures with the average of the T21 pictures weakly and strongly typical, and thus tested for feature-based stereotyping *between* categories. The second compared the T21 pictures weakly typical with those strongly typical, and thus tested for feature-based stereotyping *within* the T21 category.

The first contrast revealed a significant difference for each source of evaluation (*p*s<.05), indicating that the difference favoring positive traits at the explicit level was always smaller for T21 pictures than for TD pictures. The second contrast was significant for every source of evaluation (*p*s<.05) except one. Both student and non-student adults made slightly, but significantly, less positive judgments for the pictures strongly typical of T21 than for the weakly typical pictures. This subtle bias was not found among caregivers, for whom the T21 pictures received the most positive ratings. The target by source of evaluation interaction for the positive and negative traits taken separately is described in Text S3.

### Implicit Stereotyping (IAT)

IAT scores were calculated following the scoring algorithm recommended by Greenwald et al., including the following features: (a) error trials were removed and replaced with the block mean +600 ms, (b) response latencies >10,000 ms were removed, (c) standard deviations were calculated on all correct response trials, (d) participants who had >10% of trials with responses below 300 ms were removed, and (e) participants who had an error rate of >40% in any of the four combined sorting blocks were also removed [32]. Overall 6 participants were excluded (their inclusion in the statistical analyses did not change the results). Likewise, measures of association strength based on the two IATs were computed using the classic *D* measure [32]. *D* was the difference between the mean latencies for the “T21 pictures+positive traits” and “TD pictures+negative traits” block on one hand, and the “T21 pictures+negative traits” and “TD pictures+positive traits” block on the other hand, divided by the inclusive standard deviation of the latencies in the two blocks.

**Figure 2:**
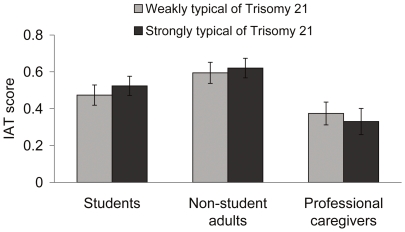
Implicit judgments. Mean IAT scores (±1 SEM) based on pictures weakly and strongly typical of T21, as a function of source of evaluation (students, non-student adults, and professional caregivers).

The *D* scores were then analyzed using a mixed ANOVA with the two IATs (based on faces either weakly or strongly typical of T21) as repeated measures, and source of evaluation (students, non-student adults, professionals) as a between-participants factor. This analysis yielded a main effect of source of evaluation, *F*(2, 162) = 6.00, *p* = .003, *η^2^*_p_ = .07 (see Figure 2); the target effect, and the source-by-target interaction were not significant (*ps*>.40). The global IAT effect (two IAT-*D* scores averaged) was smaller among the caregivers than among students and non-student adults (Tukey contrast, *p*<.05). As indicated by one-sample t-tests against zero, the global IAT effect was still clearly significant for each source of evaluation: students, *t*(54) = 10.44; non-student adults, *t*(54) = 13.08; and caregivers, *t*(54) = 5.91 (all *p*s<.001). Correlations between implicit and explicit evaluations were rather weak (see Text S4).

## Discussion

Research into the social perception of intellectually disabled persons has been largely one-sided, focusing predominantly on thoughts and feelings, which may be distorted by social desirability concerns. In the current research, we found evidence of subtle forms of prejudice toward children with special needs at both the explicit and implicit levels, in this case children with T21.

In line with previous research, students, non-student adults, and professional caregivers all attributed more positive than negative personality traits to children with T21, as they also did for TD children. However, in accordance with standard models of stereotyping operating *between* categories [20]–[23], this difference in favor of positive traits was lower for children with T21 than for TD children. Perhaps more importantly, as expected from research on feature-based stereotyping operating *within* categories [25], [26], the difference favoring positive traits proved smaller for pictures strongly typical of T21 than for weakly typical ones, at least among students and non-student adults. This is the first evidence that facial features distinctive of a genetic disorder, in this case T21, can lead to stereotyping in two ways, between and within categories. Feature-based stereotyping within the category of T21 pictures did not operate in the professional caregivers, who were also the most positive participants toward those pictures. This is consistent with the idea that relationships with intellectually disabled persons promote positive attitudes toward them (e.g., [11], [29]).

The IAT findings indicated that, in each group of participants, the T21 pictures were automatically associated with a *negative* valence. Participants, professional caregivers included, were indeed faster at categorizing T21 pictures with negative traits than T21 pictures with positive traits (and faster at categorizing TD pictures with positive traits than TD pictures with negative traits). The IAT effect also occurred regardless of whether the children's faces were strongly or weakly distinctive of T21. Taken together, these findings can be regarded as evidence that negative attitudes about, and stereotyping of, children with T21 in fact prevail at the implicit level.

Finally, the fact that the overall IAT effect was clearly significant in the three groups of participants suggests that professional caregivers' implicit mind-set about children with T21 may not differ from that of non-professionals. One important point must be made here, however. Although each group of participants exhibited a large IAT effect, this effect was significantly smaller in the caregivers than in the students and non-student adults (with equal statistical power). This suggests that caregivers' sustained contact with individuals with T21 may operate somewhat at the implicit level. Consistent with this idea, the caregivers' overall IAT effect decreased significantly with years of professional experience (which ranged from 1 to 34 years), *ß* = −.32, *t*(51) = −2.36, *p*<.03. This additional finding strengthens the conclusion of a few recent studies indicating a beneficial effect of intergroup contact on implicit evaluations (e.g., [33]). It offers a new reason to believe that even implicit stereotypes (not just explicit ones) can be reduced under the influence of repeated contact with the stigmatized. This critical issue is attracting more and more attention in the struggle against intergroup prejudice (e.g.; [34], [35]). Henry et al. showed that intergroup contact is more likely to have a beneficial impact at the implicit level in low-status groups (e.g., Blacks toward Whites but not Whites toward Blacks) [34]. Our own findings suggest that this impact can also be found in high-status groups, at least when rivalry with the low-status group is not an issue (here non-disabled people with intellectually disabled people).

Thus, the present research reveals that facial features associated with a genetic disorder such as T21 can lead to between- and within-category stereotyping at the explicit level. Although positive evaluations are especially likely at this level, they were reduced when the individuals being rated had facial features highly distinctive of this chromosomal disorder. Our research also reveals that the positive evaluations of children with T21 that can be found at the explicit level can coexist with negative evaluations at the implicit level, which helps explain why the general population is so ambivalent about practical issues such as inclusive education for these children [11], [36]. Taken together, the present findings show how important it is to pay attention to feature-based stereotyping at the explicit level. They also suggest going beyond explicit evaluations when attempting to estimate the true extent of stigmatization of intellectually disabled people. Explaining why implicit stereotyping did not operate within the T21 category is beyond the scope of this paper. However, we suggest that within-category stereotyping cannot be ruled out at this level. As noted earlier, participants in Blair et al research seemed unaware of, and unable to control, stereotyping based on Afrocentric features [26]. Thus, future research is needed to clarify whether feature-based stereotyping can occur at both the explicit and implicit levels.

A limitation of the present study (as well as many other IAT studies) is that the negative associations found at the implicit level do not necessarily mean that children with T21 are the object of implicit prejudice. As suggested earlier in this paper, however, our IAT findings are consistent with the ambivalence of the general population toward inclusive education for students with T21 [11]. Likewise, there is evidence that implicit attitudes can unintentionally lead to discriminatory behaviors such as reducing one's interaction time with the stigmatized [37], [38]. There is also evidence of discriminatory behaviors based on facial features [25]. Additional research, therefore, is also needed to determine whether the implicit attitudes and/or the visibility of the physical stigmata associated with T21 or other disorders influence the way the stigmatized are treated during social interaction. Whether those factors can raise the awareness of the disorder in the stigmatized themselves also merits special attention. People with T21 who have mental ages of about 8 years or more engage in social comparisons with other people, and start to form complex social categories of T21/disability [39]–[41]. Not only may these individuals be aware of their differences with other people, but their stigmatized identity may actually be chronically accessible, especially when their physical stigmata cannot be easily hidden. Like many other people living with a visible stigmatized identity, persons with salient phenotypic T21 features may also suffer from specific threats, for example, the threat of being judged and treated stereotypically on the basis of their facial features (at least those with mental ages above 7–8 years). This threat may contribute to lowering their performance in test situations, in the same way children and adults may suffer from stereotype threat in test situations due to their gender (e.g., [42]–[45]), age (e.g., [46]), social or academic background (e.g. [47]–[49]), or ethnic identities (e.g., [50]). Future research on these issues could help increase our understanding of stigmatization and its related consequences among people with intellectual disabilities.

Finally, one may wonder whether the negative associations found here at the implicit level are specific about T21 or could as well be explained by general implicit attitudes towards disabilities. The very few IAT studies in this area indeed indicate that people tend to associate words or pictures related to various physical disabilities [51]–[54] and mental illness [53], [55], with negatively connoted words. So far, however, we just did not know whether negative implicit associations could also be found towards people with intellectual disabilities. Here, we offer first evidence that persons (children) with T21, the most representative exemplar of the intellectually disabled, are the target of negative associations at the implicit level despite the positive stereotype that characterize them at the explicit level. As noted by Menolascino, the persons with T21 have long been regarded as “the Prince charming of the mentally retarded population” [56]. This positive view about people with T21 at the explicit level made the capture of negative associations at the implicit level especially informative. In addition, contrary to other exemplars of the intellectually disabled with known aetiology (e.g., Williams syndrome, Fragile X), persons with T21 are the only ones who can easily and automatically be identified as intellectually disabled by the general population, because they share well-known physical stigmata. This additional characteristic offered the possibility (also neglected to date) to test whether intellectually disabled people are subject to stereotyping based on their facial features (i.e., featured-based stereotyping operating within the category of the intellectually disabled). Future research based on direct comparisons between physical and intellectual disabilities might help clarify whether featured-based stereotyping can also be found for people with physical disabilities (using several degrees of physical disability), and whether the type of disability (physical vs. intellectual) makes any difference on the size of explicit and implicit attitudes.

## Supporting Information

Text S1 **Pictures of children's faces.**
(DOC)

Text S2 **Positive and negative traits.**
(DOC)

Text S3 **Target by source-of-evaluation interaction for the positive and negative traits taken separately.**
(DOC)

Text S4 **Correlations between implicit and explicit evaluations.**
(DOC)

Table S1 **Design of the two IATs.**
(DOC)
