# Supplementary material for: Tracking Subtle Stereotypes of Children with Trisomy 21: From Facial-Feature-Based to Implicit Stereotyping
Source: PLoS One. 2012 Apr 4;7(4):e34369. doi: 10.1371/journal.pone.0034369 (PMC3319569; doi:10.1371/journal.pone.0034369)
Supplement: Text S1 — Pictures of children's faces. (DOC) [file pone.0034369.s001.doc]

Text S1

**Pictures of children faces**

The pictures used in the present research were selected based on a preliminary study with another subject sample made up of students (*N* = 111). We collected ratings to determine how 30 photographed faces of children, 7-14 years old, with (20) or without T21 (10), were perceived with regard to this disorder. Participants rated whether the faces were typical of T21 using Likert scales ranging from 1 (no T21 features) to 5 (all T21 features), and so could be considered “raters” or “judges” of T21 (i.e., 111 judges of 30 faces). We computed an intraclass correlation coefficient (*ICC*) to examine rater reliability [1], and found the ratings to be reliable (*ICC* =.98). We then used the mean rating of each face as our measure of face typicality regarding T21, and selected 12 pictures on this basis (6 rated as weakly typical and 6 as strongly typical).

Reference

1. Shrout, PE., Fleiss, JL (1979) Intraclass correlations: Uses in assessing rater reliability. Psychol Bull 86: 420-428. doi:10.1037/0033-2909.86.2.420
