# Supplementary material for: Tracking Subtle Stereotypes of Children with Trisomy 21: From Facial-Feature-Based to Implicit Stereotyping
Source: PLoS One. 2012 Apr 4;7(4):e34369. doi: 10.1371/journal.pone.0034369 (PMC3319569; doi:10.1371/journal.pone.0034369)
Supplement: Text S2 — Positive and negative traits. (DOC) [file pone.0034369.s002.doc]

Text S2

**Positive and negative traits**

The traits used in the explicit and implicit tasks consisted of 6 positive traits (“humain” [humane], “affectueux” [affectionate]”, “éducable” [educable], “attachant” [endearing], “sociable” [sociable/friendly], and “intelligent” [intelligent]), and 6 negative traits (“méchant” [mean], “bête” [stupid], “laid” [ugly] “gênant” [annoying], “agressif” [aggressive], and “déformé” [deformed]). Cronbach’s alpha was .94 for both the 6 positive and the 6 negative traits. These traits were selected from a preliminary study where 244 participants indicated whether each of 47 traits (generated by previous participants) may apply to children with T21, using a 6-point Likert scale ranging from 1 (strongly disagree) to 6 (strongly agree).
