# Supplementary material for: Tracking Subtle Stereotypes of Children with Trisomy 21: From Facial-Feature-Based to Implicit Stereotyping
Source: PLoS One. 2012 Apr 4;7(4):e34369. doi: 10.1371/journal.pone.0034369 (PMC3319569; doi:10.1371/journal.pone.0034369)
Supplement: Text S3 — Target by source-of-evaluation interaction for the positive and negative traits taken separately. (DOC) [file pone.0034369.s003.doc]

Text S3

**Target by source-of-evaluation interaction for the positive and negative traits taken separately**

The target by source-of-evaluation interaction was also significant for the positive and negative traits taken separately (*Fs* = 9.33 and 6.18, *ps* <. 001, *η^2^*_p_ = .10 and .07, respectively). The same two Helmert contrasts as those calculated on the difference scores (TD pictures vs. T21 pictures; T21 pictures weakly typical vs. strongly typical) also yielded significant differences for each source of evaluation. For the three groups of participants, TD pictures were rated more positively and less negatively than T21 pictures. The findings of the second contrast were more complex. Among the students and non-student adults, the pictures weakly typical of T21 were rated more positively and less negatively than those strongly typical. Among the professionals caregivers, the pictures *strongly* typical of T21 were evaluated *more* positively, and simultaneously more negatively, than those weakly typical. It is worth noting that students and non-student adults produced more variable judgments than the professionals especially when rating faces strongly typical of T21. This is consistent with the social relation model [1] that predicts more consensual judgments when the perceivers (professionals) are more familiar with the targets (T21).

Reference

1. Kenny, DA, Albright, L (1987). Accuracy in interpersonal perception: a social relations analysis. Psychol Bull 102:390-402.
