# Supplementary material for: Tracking Subtle Stereotypes of Children with Trisomy 21: From Facial-Feature-Based to Implicit Stereotyping
Source: PLoS One. 2012 Apr 4;7(4):e34369. doi: 10.1371/journal.pone.0034369 (PMC3319569; doi:10.1371/journal.pone.0034369)
Supplement: Text S4 — Correlations between implicit and explicit evaluations. (DOC) [file pone.0034369.s004.doc]

Text S4

**Correlations between implicit and explicit evaluations**

The relationship between implicit and explicit evaluations is generally weak (e.g., [1]). Consistent with this, in each group of participants, the correlations between the explicit and implicit evaluations were rather weak and not always significant (the higher the difference favoring the positive traits, the lower the IAT score: -.39, *p* < .003; -.20, *p* = .14; -.32, *p* < .02; for students, non-student adults, and caregivers, respectively).

Reference

1. Hofmann, W, Gawronski, B, Gschwendner, T, Le, H, Schmitt, M (2005) A meta-analysis on the correlation between the Implicit Association Test and explicit self-report measures. Pers Soc Psychol B 31:1369-1385. doi :10.1177/0146167205275613
