# Supplementary material for: Tracking Subtle Stereotypes of Children with Trisomy 21: From Facial-Feature-Based to Implicit Stereotyping
Source: PLoS One. 2012 Apr 4;7(4):e34369. doi: 10.1371/journal.pone.0034369 (PMC3319569; doi:10.1371/journal.pone.0034369)
Supplement: Table S1 — Design of the two IATs. (DOC) [file pone.0034369.s005.doc]

**Table S1:** Design of the two IATs

| Block | Function | Type of stimuli | Left key | Right key | No. of trials |
| --- | --- | --- | --- | --- | --- |
| 1 | Practice | Pictures | Normal | Trisomy | 24 |
| 2 | Practice | Words | Positive | Negative | 24 |
| 3 | Practice | Pictures + Words | Normal + Positive | Trisomy + Negative | 24 |
| 4 | Test (Congruence) | Pictures + Words | Normal + Positive | Trisomy + Negative | 96 |
| 5 | Practice | Pictures | Trisomy | Normal | 24 |
| 6 | Practice | Pictures + Words | Trisomy + Positive | Normal + Negative | 24 |
| 7 | Test (Incongruence) | Pictures + Words | Trisomy + Positive | Normal + Negative | 96 |
